# Nanostructured Strategies for Melanoma Treatment—Part I: Design and Optimization of Curcumin-Loaded Micelles for Enhanced Anticancer Activity

**DOI:** 10.3390/ph18030327

**Published:** 2025-02-26

**Authors:** Valentina Paganini, Andrea Cesari, Silvia Tampucci, Patrizia Chetoni, Susi Burgalassi, Michele Lai, Giulia Sciandrone, Silvia Pizzimenti, Fabio Bellina, Daniela Monti

**Affiliations:** 1 Department of Pharmacy, University of Pisa, Via Bonanno 33, 56126 Pisa, Italy; valentina.paganini@phd.unipi.it (V.P.); patrizia.chetoni@unipi.it (P.C.); susi.burgalassi@unipi.it (S.B.); silvia.pizzimenti@unipi.it (S.P.); daniela.monti@unipi.it (D.M.); 2 Department of Chemistry and Industrial Chemistry, University of Pisa, 56124 Pisa, Italy; andrea.cesari@unipi.it (A.C.); fabio.bellina@unipi.it (F.B.); 3 Italian Inter-University Center for the Promotion of the 3Rs in Teaching and Research, University of Pisa, 56122 Pisa, Italy; 4 Retrovirus Center and Virology Section, Department of Translational Research and New Technologies in Medicine and Surgery, University of Pisa, 56100 Pisa, Italy; michele.lai@unipi.it (M.L.); giulia.sciandrone@gmail.com (G.S.)

**Keywords:** curcumin, drug delivery systems, nanotechnology, micelles, melanoma skin cancer, TPGS, response surface methodology (RSM), high-content imaging system

## Abstract

**Background/Objectives**: Melanoma is a pathology that affects a large part of the population, and the currently available therapies have many limitations, including the selective targeting of the site of action. This study explores the development of curcumin (CUR)-loaded nanostructured delivery systems for topical melanoma treatment, addressing CUR’s limitations in bioavailability, solubility, and stability. **Methods**: Binary surfactant mixtures of Vitamin E-TPGS (TPGS) and Kolliphor ELP (ELP) were selected to form stable micelles for curcumin encapsulation. A Design of Experiments (DoE) approach was applied to optimize the surfactant ratios for enhanced drug solubilization and improved cytotoxic effects on melanoma cells. The final formulation was characterized using Fourier Transform Infrared Spectroscopy (FTIR), Differential Scanning Calorimetry (DSC), and Nuclear Magnetic Resonance (NMR) spectroscopy to confirm its properties. **Results**: The final formulation, TPGS30ELP15, contained 30 mM TPGS and 15 mM ELP and led to formation of nanostructures of the expected size (hydrodinamic diameter, Dh: 13.11 ± 0.01 nm; polydispersivity index, PDI = 0.371 ± 0.05), able to solubilize 5.51 ± 1.09 mM CUR. The formulation was stable for a 120-day period stored at 4 °C and room temperature in the dark. Cytotoxicity testing in A375 melanoma cells demonstrated that curcumin-loaded micelles significantly reduced cell viability compared to free curcumin. Long-term exposure (24 h) revealed that free curcumin caused an 85% reduction in cell viability, while TPGS30ELP15 resulted in a 70% reduction. Additionally, free curcumin induced a 30% increase in cytoplasmic area, indicating necrosis, whereas TPGS30ELP15 decreased the cytoplasmic area by 20%, suggesting apoptosis. **Conclusions**: This study demonstrates that TPGS30ELP15 nanomicelles enhance curcumin’s anticancer effects while promoting apoptosis and minimizing necrosis, which is associated with lower inflammation and tissue damage. These findings suggest that TPGS30ELP15 offers a more favorable therapeutic profile for melanoma treatment, paving the way for safer and more effective topical therapies.

## 1. Introduction

Skin cancer is a malignancy with a globally rising incidence. Among the various types of skin cancer, melanoma is particularly concerning, as it accounts for the majority of skin cancer-related fatalities. Although surgical intervention remains the primary treatment modality, non-surgical adjuvant therapies are being increasingly explored. However, traditional chemotherapy often leads to severe adverse effects and the development of multi-drug resistance (MDR) [1]. Consequently, the exploration of novel therapeutic strategies, including targeted drug delivery systems and natural compounds with pharmacological properties, is imperative [2].

Curcumin (CUR), a yellow pigment derived from the rhizome of *Curcuma longa*, is a natural compound recognized for its diverse pharmacological properties, including anti-inflammatory, antimicrobial, anticancer, and antioxidant activities [3,4,5,6]. Additionally, the US Food and Drug Administration (USFDA) classifies CUR as “Generally Recognized As Safe” (GRAS) [7]. Despite its therapeutic potential, the clinical application of CUR is severely limited by its instability, poor solubility, and low permeability, classifying it as a Biopharmaceutics Classification System (BCS) Class IV drug [4]. These limitations necessitate the development of innovative delivery systems to harness its full therapeutic benefits.

Over the past decade, various pharmaceutical approaches have been investigated to enhance CUR’s bioavailability, including nanoparticles, liposomes, micelles, and solid dispersions [8,9]. Topical administration has emerged as a promising strategy, particularly for melanoma treatment, as it enables the delivery of higher drug concentrations directly to the tumor site, while minimizing systemic exposure and associated side effects [10,11]. Recent studies have shown that CUR induces apoptosis in melanoma cells and inhibits metastasis, making it a valuable candidate for topical formulations targeting skin cancer [12,13]. However, CUR’s physicochemical properties pose significant challenges for effective topical delivery.

CUR undergoes keto-enol tautomerism, existing predominantly in the enol form in alkaline aqueous solutions and as a keto form in acidic and neutral conditions. While this tautomerism contributes to its pharmacological activity, it also results in instability, leading to degradation in neutral or alkaline aqueous environments [14]. Furthermore, exposure to ultraviolet-visible light exacerbates curcumin degradation, complicating the formulation of stable topical applications [15].

Nanostructured drug delivery systems offer a promising solution to these challenges by improving curcumin’s solubility, stability, and target-site concentration [16,17,18,19]. Such systems also enhance skin penetration, enabling higher local drug concentrations while reducing systemic exposure [20].

Studies have demonstrated that CUR-loaded polymeric nanoparticles exhibit superior cytotoxicity against melanoma cells compared to free curcumin [21]. Additionally, CUR-loaded lipid nanoparticles have been shown to improve skin penetration and antitumor efficacy in melanoma models [22,23]. These findings highlight the potential of nanostructured delivery systems to overcome CUR’s limitations and optimize its therapeutic efficacy.

The current study aimed to develop CUR-loaded micelles for topical application in melanoma treatment. The nanomicellar system was chosen for its simple preparation method (direct dissolution), where water-soluble polymers or surfactant macromolecules spontaneously form micelles above the critical micellar concentration, encapsulating the active ingredient within the core. This approach offers better scalability compared to other nanocarriers that require more complex, lengthy, and costly production processes. The micelles’ small size enhances passive targeting of solid tumors, even those with limited permeability, and promotes cellular internalization, particularly in melanoma cells [24]. Additionally, the amphiphilic polymers used in micelle formation interact with biological membranes, further enhancing drug delivery and stability.

The tested formulations consisted of binary surfactant mixtures designed to solubilize curcumin in a hydrophilic environment and protect it from degradation. Surfactants were carefully chosen based on their ability to form stable micelles, safety, and biocompatibility. Vitamin E-TPGS (TPGS) and Kolliphor ELP (ELP) were identified as the optimal surfactants.

A design of experiment (DoE) approach was employed to evaluate the effect of surfactant ratios on drug solubilization and melanoma cell viability. Advanced physicochemical characterization techniques, including FTIR, DSC, and NMR spectroscopy, were utilized for the physicochemical characterization of the formulation. In this context, NMR represents a versatile technique for the monitoring of surfactant-assisted nanoparticles synthesis [25], detailed descriptions of guest–host inclusion complexes [26], and characterization of drug delivery systems [27,28].

Stability studies conducted under varying light and temperature conditions demonstrated the robustness of the nanomicellar formulations, effectively addressing CUR degradation in aqueous solutions.

Finally, the cytotoxic effects of the CUR-loaded micelles were assessed in melanoma cell lines using the Operetta CLS high-content imaging system, confirming their efficacy in targeting melanoma cells.

This study highlights the advantages of scalable, stable, and efficient nanostructured systems for CUR delivery, offering substantial contributions to the development of topical therapies for melanoma. By effectively addressing key challenges associated with CUR, such as its poor bioavailability and instability, nanomicellar formulations emerge as a promising approach to enhance therapeutic outcomes and improve patient care. Furthermore, these findings advance the field of targeted drug delivery for skin cancer treatment, demonstrating the potential of these systems to provide safer and more effective melanoma therapies compared to existing methods.

## 2. Results and Discussion

### 2.1. Design of Experiment (DoE) Study for the Selection of a Curcumin-Loaded Nanomicellar Formulation Based on a Binary Mixture of Surfactants

This research aimed to develop a nanomicellar delivery system optimized for size, size distribution, drug stability, and effective curcumin delivery to the target site (the skin), with minimal side effects. A pre-formulative study, coupled with a Design of Experiments (DOE) approach, was employed to evaluate the influence of surfactant ratios on drug solubilization efficacy and melanoma cell viability. Developing micelles based on a binary mixture of surfactants could ensure the thermodynamic stability of the system post-administration. Combining two or more surfactants reduces the critical micellar concentration (CMC), thereby improving the stability of the nanomicellar formulation upon dilution with biological fluids [29].

The selected surfactants were d-α-tocopherol polyethylene glycol succinate (TPGS) and polyoxyl-35-castor oil (ELP). TPGS, a water-soluble derivative of natural vitamin E, is synthesized by esterifying vitamin E succinate with polyethylene glycol 1000. It is widely used as emulsifier, solubilizer, and permeation enhancer. TPGS is a non-ionic surfactant with a lipophilic/hydrophilic balance (HLB) and CMC values of 13.2 and 0.02% w/w, respectively. Beyond its CMC, TPGS forms small micelles (size above 15 nm), which are particularly advantageous for targeting cancer cells. Smaller micelles exhibit enhanced cellular internalization, thereby improving drug permeation and retention within cancerous tissues [30,31].

Additionally, TPGS has demonstrated the ability to inhibit P-glycoprotein (P-gp), an ATP-dependent efflux pump involved in multidrug resistance (MDR) in cancer cells. By limiting P-gp-mediated efflux, TPGS enhances intracellular drug accumulation, potentiating the cytotoxicity of chemotherapeutic agents. Furthermore, TPGS exhibits antitumor properties, inducing apoptosis and synergizing with other anticancer drugs. It selectively induces cytotoxic effects on cancer cells through three main mechanisms: inhibition of mitochondrial respiratory complex II with concurrent reactive oxygen species (ROS) generation, induction of DNA damage, and downregulation of anti-apoptotic proteins (Survivin and Bcl-2) [32]. Importantly, the United States Food and Drug Administration (FDA) has approved TPGS as a safe pharmaceutical adjuvant for incorporation in drug formulations.

ELP, another non-ionic surfactant included in this study, has a CMC of 0.02% w/w and exhibits strong solubilization potential in aqueous systems. This surfactant is particularly well-suited for stabilizing sensitive active pharmaceutical ingredients (APIs) and is compatible with a wide range of excipients. Similar to TPGS, ELP forms small micelles (Technical data sheet, BASF) and can modulate P-gp activity, thereby enhancing cellular drug absorption by inhibiting P-gp-mediated efflux [33].

CUR micelles were prepared by mixing different molar ratios of the selected surfactants, comparing the results obtained with those of the single surfactant at the same molar concentration used in the binary mixture. The results of the characterization of the micelles are reported in Table 1, in which the tested formulation is indicated by the name of the surfactant followed by the number representing its mM concentration.

All formulations are nanomicellar dispersions with a hydrodynamic diameter ranging from 11.47 to 13.79 nm with polydispersivity indexes in the range 0.345–0.70. The nanomicelles developed in this study, with an average size of 13 nm and a PDI of 0.3, are notably smaller than those reported in the literature for curcumin-loaded nanostructured delivery systems, which typically range from 10 to 100 nm [34].

Such a remarkably small size offers significant advantages in terms of tissue penetration and biodistribution. The PDI value of 0.3, while slightly above the threshold for a monodisperse system (<0.2), remains within an acceptable range for nanotherapeutic applications, indicating a reasonable degree of size uniformity. This characteristic could positively influence colloidal stability and therapeutic performance. Overall, the exceptional small size of these nanomicelles differentiates them from many reported systems and presents promising potential for enhanced bioavailability and therapeutic efficacy of curcumin [35,36]. Further studies are warranted to investigate the impact of these features on biological performance.

#### 2.1.1. Comparison of Model Complexity for Predicting Curcumin Solubilization and Cell Viability in Melanoma Cell Lines (A375)

The experimental data on the solubilized curcumin (CUR) concentration and cell viability were used to build a DoE study. This approach facilitated the determination of the optimal molar ratio of the two surfactants under investigation, TPGS and ELP. The primary objective of this study was to accurately describe the phenomena of CUR solubilization and cell viability. Moreover, the study aimed to generate predictive insights into the optimal combination of surfactants that would maximize CUR solubilization efficiency while simultaneously achieving the highest cytotoxic effect against melanoma cells.

This methodical analysis not only provided a detailed understanding of the interaction dynamics between CUR and the surfactant system but also highlighted the synergistic potential of TPGS and ELP in enhancing CUR’s therapeutic efficacy.

To achieve this objective, models of varying complexity were evaluated to identify the most suitable fit for the experimental data. The outcomes of these assessments are presented in Table 1.

A quadratic model was initially employed to capture the potentially complex interactions between the two surfactants, TPGS and ELP, on CUR solubilization and cell viability. Despite the limited number of observations (n = 9), this approach aimed to adequately describe the intricate interactions between the variables. For CUR solubilization, the model demonstrated a strong fit, with high R-squared (R^2^) and adjusted R-squared (adj R^2^) values, 0.941 and 0.843 respectively, suggesting a good fit. Conversely, for cell viability, there was a substantial difference between R^2^ (0.700) and adj R^2^ (0.200), indicating potential overfitting and a poor model fit. The coefficients for the quadratic terms in both models were very close to zero (Equations (1) and (2)), highlighting the limited significance of the quadratic components. This suggests that the quadratic terms may not contribute meaningfully to the model, raising concerns about the model’s complexity relative to the data size. For CUR solubilization, the quadratic model produced an F-statistic of 9.62 with a *p*-value of 0.0458. This suggests that the model fits the data reasonably well; however, the statistical significance is marginal, being close to the 0.05 threshold, therefore limiting the robustness of this conclusion. In contrast, the quadratic model for cell viability exhibited an F-statistic of 1.4 and a *p*-value of 0.416, indicating a lack of statistical significance. This suggests that the quadratic terms fail to adequately explain the variability in the cell viability data, likely due to overfitting and the insufficiency of the dataset.

(1) $$\begin{array}{l} CUR\;solubilization\\ \;=1.399+0.08939\;TPGS+0.07068\;ELP+0.0008229\;TPGSELP-0.0002045\;{TPGS}^{2}\\ \;+0.002365\;{ELP}^{2} \end{array}$$

(2) $$\begin{array}{l} Cell\;viability=90.5-1.087\;TPGS+1.301\;ELP+0.03708\;TPGSELP-0.001931\;{TPGS}^{2}\\ \;-0.05672\;{ELP}^{2} \end{array}$$

Thereafter, a linear interaction model was tested. This model produced high R^2^ and adj R^2^ values (Table 2), which were much closer to each other compared to the quadratic model, suggesting a better balance between model complexity and fit. For CUR solubilization, this model (Equation (3)) yielded an F-statistic of 24.4 and a *p*-value of 0.00206, indicating a significant and robust model. Importantly, this model highlighted the significance of TPGS (*p*-value 0.023854). For cell viability, the same linear interaction model (Equation (4)) produced an F-statistic of 3.44 and a *p*-value of 0.109, indicating a trend toward significance but not reaching the conventional threshold. This suggests that while the model may capture some relevant interactions, it is not as robust in predicting cell viability as it is for solubilization.

(3) CUR solubilization=1.2212+0.084836 TPGS+0.12553 ELP+0.0006039 TPGSELP

(4) Cell viability=96.84−1.2758 TPGS−0.0816 ELP+0.043888 TPGSELP

Finally, a simplified linear model was evaluated. While this model resulted in slightly lower R^2^ and adjusted R^2^ values, particularly for the cell viability outcome, the reduced discrepancy between these metrics suggested a decreased risk of overfitting. This simplification provided more consistent and interpretable results across both response variables. For CUR solubilization, this model (Equation (5)) yielded an F-statistic of 42.9 and a *p*-value of 0.00028, indicating a significant and robust model, which highlighted the significance of both independent variables (TPGS and ELP, *p*-values 0.00019537 and 0.00072271, respectively) in the solubilization process. In contrast, for cell viability, the same linear model (Equation (6)) yielded an F-statistic of 5.0 and a *p*-value of 0.0527. Although this *p*-value approaches the conventional threshold for statistical significance, it does not meet the criterion, suggesting that the model’s explanatory power for cell death is less robust.

(5) CUR solubilization=1.0079+0.092683 TPGS+0.14104 ELP

(6) Cell viability=81.651−0.64229 TPGS+0.94624 ELP

The results obtained from the statistical analysis of the models are summarized in Table 2.

Based on these analyses, the linear model was selected as the most suitable fit for our data, offering an optimal balance between explanatory power and simplicity, particularly in the contest of solubilization outcomes. However, the cell viability results indicate that additional factors or a larger dataset may be necessary to fully elucidate the interactions affecting cell death induction.

#### 2.1.2. Analysis of the Response Surfaces Computed with the Selected Linear Models

**Figure 1:**
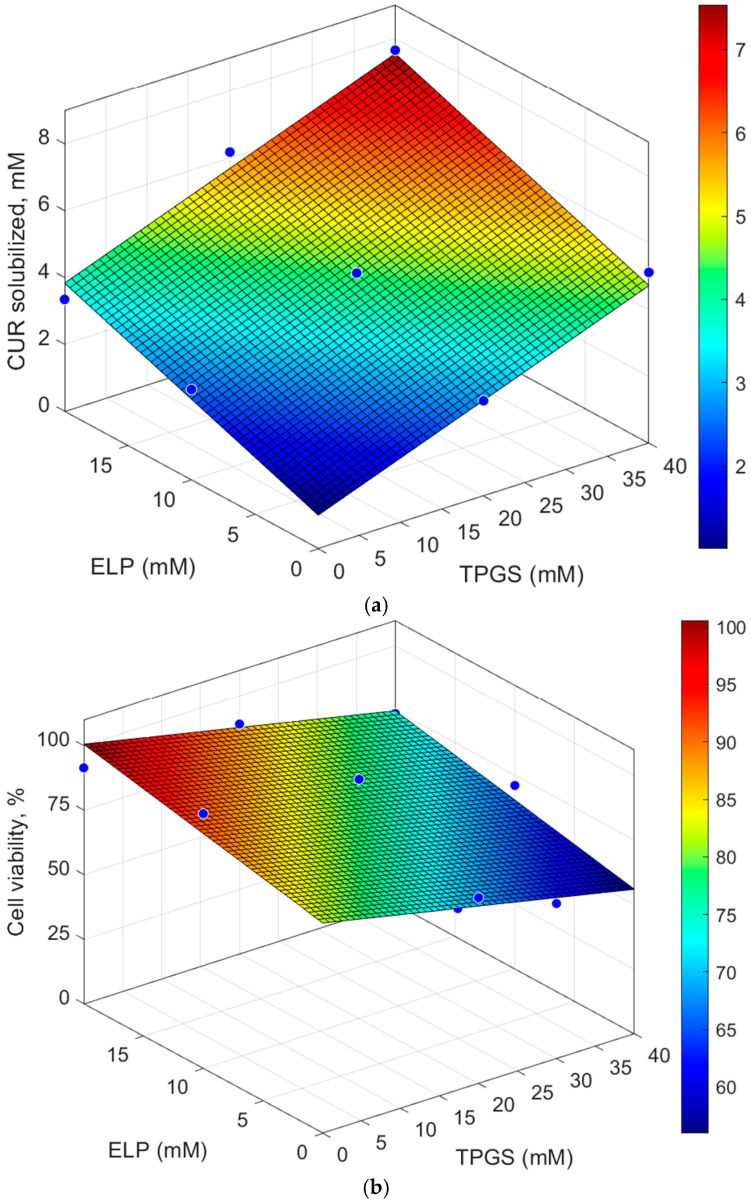
Response surfaces for the two dependent variables: CUR solubilized (**a**) and Cell viability (**b**). Blue dots represent experimental values.

The linear models for both CUR solubilization and cell death were employed to generate response surfaces, which facilitated the identification of the optimal molar ratios for the surfactants TPGS and ELP. Although the cell viability model did not reach statistical significance, it provided valuable insights into the system’s behavior. The results of the Design of Experiments (DOE) surface analysis illustrated in Figure 1 depict the relationships between the two surfactants and their effect on CUR solubilization (mM) (a) and cell viability (b).

For CUR solubilization, response surface analysis revealed that both TPGS and ELP concentrations significantly impacted drug solubility, as demonstrated from the ANOVA analysis. CUR solubilization appears to correlate with the total amount of surfactants in the system: higher total surfactant content results in greater quantities of solubilized CUR. Optimal solubilization was achieved with TPGS concentrations exceeding 20 mM and ELP concentrations above 15 mM. The highest amount of solubilized CUR (7.64 mM) was achieved with a total surfactant content of 60 mM, obtained through the combination of 40 mM TPGS and 20 mM ELP (ratio 2:1, TPGS40ELP20), followed by TPGS20ELP20 (solubilized CUR:6.17 mM, total surfactant content: 40 mM), TPGS40ELP10 (solubilized CUR: 5.57 mM, total surfactant content: 50 mM), TPGS20ELP10 (solubilized CUR: 4.61 mM, total surfactant content: 30 mM) and TPGS25ELP5 (solubilized CUR: 3.47 mM, total surfactant content: 30 mM) formulations. A similar trend was observed in terms of encapsulation efficiency, as percentage of drug entrapped with respect to the surfactant total amount added during the micelle preparation.

In terms of cell viability, the response surface analysis indicated that the optimal concentration of TPGS was greater than 20 mM, while the most favorable results for ELP were achieved at concentrations below 15 mM. Formulations containing TPGS at both 20 and 40 mM concentrations appeared to increase cell death compared to CUR 5µM aqueous solution (reference), reducing viable cells from 89.21 ± 5.64% for CUR to 72.02 ± 4.89 and 60.11 ± 7.04% for TPGS20 and TPGS40 formulations, respectively, with significant statistically differences observed in the case of TPGS40 (*p* = 0.01). However, among the mixed micelles, the most performing formulation was the one with the highest molar ratio of TPGS to ELP (5:1). TPGS25ELP5 formulation significantly reduced cell viability compared to the reference (50.50 ± 4.85%; *p* = 0.001), suggesting that TPGS plays a pivotal role in inducing cell death. Furthermore, minimizing ELP concentrations appeared to mitigate potential cytotoxic effects.

The DoE analysis identified the TPGS30ELP15 formulation, consisting of 30 mM TPGS and 15 mM ELP (molar ratio 2:1; total surfactant concentration: 45 mM), as the most promising candidate. This formulation not only demonstrated optimal CUR solubilization, but effectively managed cell viability, making it a strong candidate for further development and detailed characterization in future studies.

### 2.2. Characterization of the Selected CUR Nanomicellar Formulation (TPGS30ELP15)

The formulation identified through the DoE analysis was prepared following the method previously reported and subsequently characterized in terms of dimensional analysis. The results confirmed the successful formation of nanostructures with the expected size (Dh: 13.11 ± 0.01 nm; PDI = 0.371 ± 0.05). Additionally, the formulation exhibited efficient solubilization capacity, achieving a solubilized CUR concentration of 5.51 ± 1.09 mM and an encapsulation efficiency (EE) of 67.72 ± 13.46% (n = 3). These findings validated the predictions made by the DoE, demonstrating the reliability and robustness of the design approach in optimizing the formulation.

#### 2.2.1. ATR-FTIR Analysis

To demonstrate the encapsulation of CUR within the TPGS30ELP315 micelles, ATR-FTIR analyses were performed to detect any structural changes.

**Figure 2:**
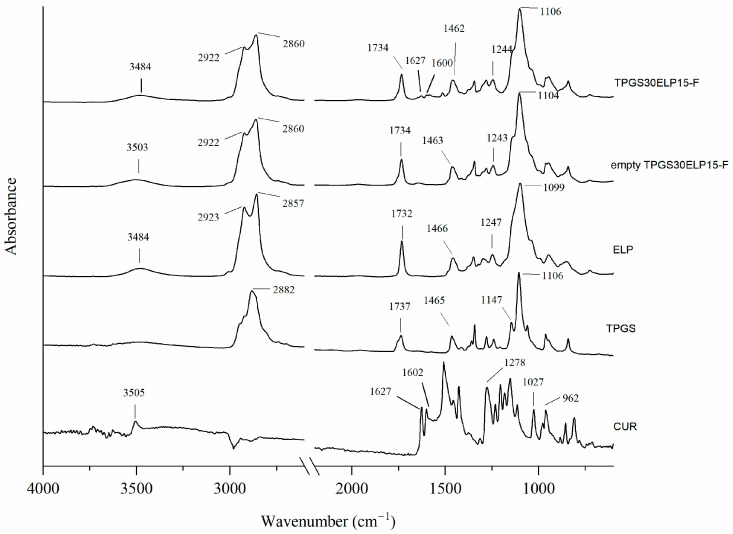
ATR- FTIR spectra of raw materials (curcumin, TPGS, ELP) and freeze-dried micelles with and without curcumin (TPGS30ELP15-F and empty TPGS30ELP15-F, respectively).

The ATR-FTIR spectra of the curcumin powder (CUR), freeze-dried micelles with and without curcumin (TPGS30ELP15-F and empty TPGS30ELP15-F, respectively) are shown in Figure 2.

The ATR-FTIR spectrum of curcumin exhibits well-known characteristic peaks that have been extensively documented in the literature [37,38]. These peaks serve as definitive markers for the identification of curcumin and its molecular structure. The band at 3505 cm^−1^ corresponds to the phenolic O–H stretching vibration characteristic of curcumin’s molecular structure. The peak at 1627 cm^−1^ is attributed to the vibrations of the C=O and C=C bonds, while the band at 1602 cm^−1^ arises from the symmetric vibration of the aromatic ring (C=C). Additionally, the band at 1508 cm^−1^ is associated with the stretching vibration of the C=O group. The band at 1278 cm^−1^ corresponds to the stretching vibration of the phenol C–O group, and the peak at 1027 cm^−1^ is indicative of the C–O–C linkage. Furthermore, the bands at 962 cm^−1^ are assigned to the benzoate trans-C–H vibration.

The ATR-FTIR spectra of TPGS and ELP exhibited characteristic bands consistent with those reported in literature [39,40]. Both surfactants displayed a band at 2884 cm^−1^, corresponding to C–H stretching, and a carbonyl band at 1735 cm^−1^. Typical C–O stretching and C–H bending vibrations were observed at approximately 1100 cm^−1^ and 1464 cm^−1^, respectively.

A comparison of the ATR-FTIR spectra of empty micelles and CUR-loaded micelles revealed that the characteristic bands of the surfactants were present in the spectrum of the empty micelles. These included the 2860 cm^−1^ band (C–H stretching), the carbonyl band at 1735 cm^−1^, the 1460–1240 cm^−1^ peak (C–O–C stretching), and the 1100 cm^−1^ band (C–O stretching).

Notably, in the spectrum of the curcumin-loaded formulation (TPGS30ELP15-F), the bands at 1627–1600 cm^−1^, corresponding to C=O and C=C stretching, were clearly observed. In contrast, these bands were absent in the spectrum of the empty micelles (empty TPGS30ELP15-F). This observation provides strong evidence that curcumin was successfully encapsulated within the nanomicellar structure.

#### 2.2.2. DSC Analysis

**Figure 3:**
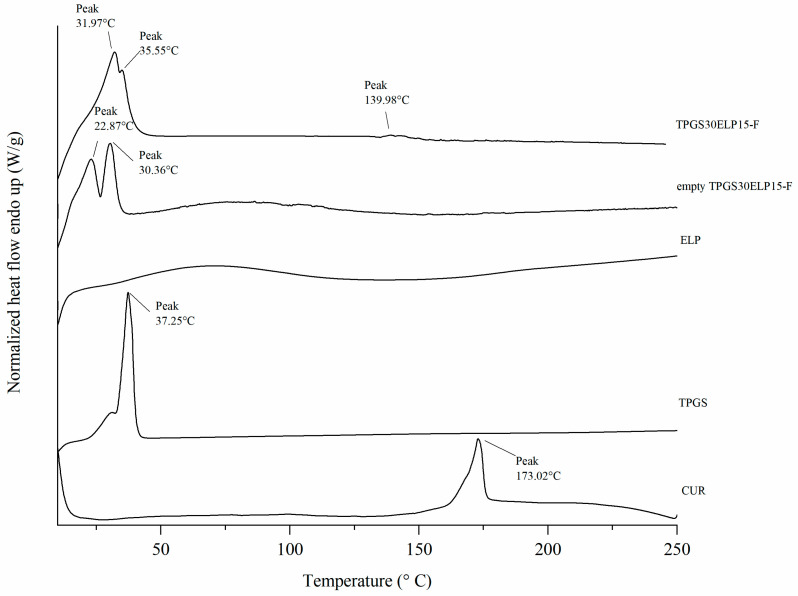
DSC thermograms of raw materials (curcumin, TPGS, ELP) and freeze-dried micelles with and without curcumin (TPGS30ELP15-F and empty TPGS30ELP15-F, respectively).

Further characterization of the curcumin-loaded nanomicelles formulation was performed by DSC, with the results presented in Figure 3. The thermogram of the curcumin-loaded micelles exhibited a signal at 140 °C, which was absent in the empty micelles. This signal is likely attributable to the encapsulated drug and is shifted compared to the melting point of raw curcumin (~173 °C). This behavior may be explained by two factors: the encapsulation of curcumin within the micellar structure, which can alter its thermal properties, and the relatively small quantity of encapsulated curcumin, which may limit the detectability of a more pronounced thermal transition.

#### 2.2.3. NMR Analysis

As a preliminary step, ^1^H NMR spectrum in concentrated solution (20 mM, DMSO-d_6_) was recorded prior to the analysis of TPGS30ELP15. DMSO-d_6_ was selected due to the highest CUR solubility in it [41], whereas CUR water solubility as reported were remarkably low (1630 nM, 30 nM, and <21 nM) [42,43,44]. The CUR spectrum is complicated by the instauration of keto-enolic equilibrium and by the presence of possible other curcuminoids (Scheme S1), likely to be monodemethoxy-curcumin (MDCUR) and bisdemethoxy-curcumin (BDCUR), the presence of which can increase CUR solubility. Keto-enol species in DMSO-d_6_ was reported to be the main component (97.4%, CH_2_ 6.05 and CH 4.12 ppm) with respect to the β-Diketone one [45]. From a low intensity overview of the ^1^H NMR spectrum of CUR (Figure S1), it was possible to recognize -OHs (9.60 ppm), aromatic (6.5–7.8 ppm), double bond (6.02 ppm), -OCH_3_ (3.80 ppm) resonances. Since the protons belonging to the central double bond of hypothesized curcuminoids were gathered at 6.0–5.8 ppm (H_1_^enol^), their resonance integrated area was set to 1. Consequentially, the methoxy integrated area could indicate the presence of 9% of BDCUR. By increasing the intensity, it was possible to observe the presence of other components with relevant features (Figure S2). Roughly, their estimation was calculated to be 20% (from peak at ~6.65 ppm). ^1^H NMR spectrum of ELP in quantitative conditions (Figure S3) confirmed the average amount of PEGylated chains (n~12.5) declared by the supplier. Also, for TPGS (Figure S4) the number of PEGylated chains was in line with what was declared (n~23.2).

**Figure 4:**
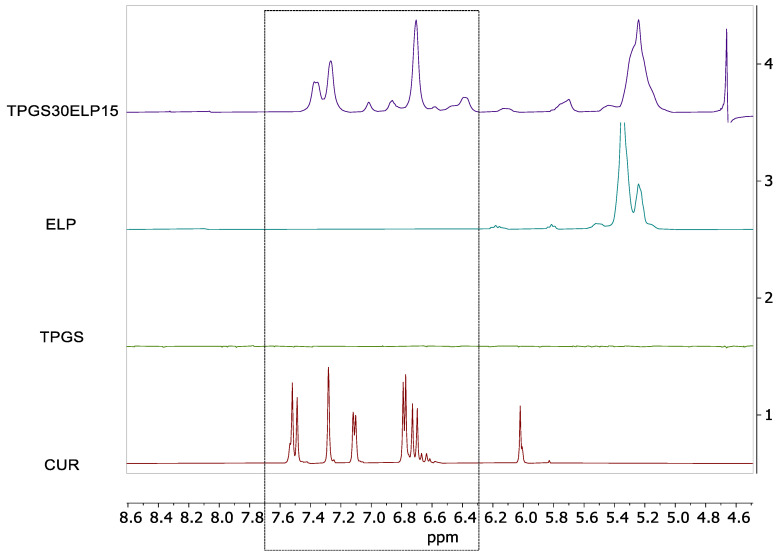
H NMR WATERGATE (500 MHz, D_2_O, 25 °C) spectra of CUR, ELP, TPGS and TPGS30ELP15.

After the identification of NMR resonance pattern of the isolated components, the micelles formulations (TPGS30ELP15) were carefully investigated. In Figure 4 (full spectra reported in Figure S5) are reported the ^1^H NMR spectra of CUR (1), ELP (2), TPGS (3) and TPGS30ELP15 (4). In the case of nano formulations, minimum sample treatment was carried out (addition of 10% of D_2_O for locking) and a water suppression sequence was used.

Additional aromatic CUR spectral resonances are clearly present in TPGS30ELP15, confirming the capacity of the nanomicellar formulation to solubilize and bind CUR. As a matter of fact, the signal of CUR underwent relevant line broadening due to the interaction with the micelles. Other near resonances (<6.4 ppm) belong to ELP and were ignored for the quantification. The ratio between molecular components constituting the nanomicellar formulation and also the mM concentration reached by CUR (total curcuminoid), and hence encapsulation efficiency, was quantified by NMR. Since in TPGS30ELP15 a water suppression was employed, a control for resonances attenuation near water peak had to be conducted, particularly for ELP double bonds and PEG resonances. Differences in the ELP integrated area with or without water suppression were negligible (~2.5%, Figure S6). For ELP, the characteristic double bonds resonances were selected, and the corresponding CH_3_^ELP^ integrated area contribution at 0.75 ppm was subtracted from the total integrated area of methyl fragments (Figure S7). The remaining CH_3_ signals were attributed to TPGS and thus the relative ratio of TPGS/ELP for TPGS30ELP15 was calculated as 32.9:15, with great accordance to theoretical values.

For CUR absolute quantification, the preparation of a quantitative standard with a sufficient concentration in D_2_O was not possible. A solution of CUR in DMSO-d_6_ (5 mM) was prepared instead and the amount of CUR was calculated in TPGS30ELP15 (in this way, all aromatic regions containing potential curcuminoid were counted as well, Figure 4). The CUR concentration thus calculated was 3.2 mM (1.2 mg/mL). This result is in line with the CUR quantification found in the UV-Vis determination. The increasing solubility of CUR in TPGS30ELP15 is thus at least three orders of magnitude with respect to pure CUR in water medium. NMR analysis confirmed the composition of the formulation both in terms of drug loading and surfactant content with respect to the CUR.

#### 2.2.4. Transmission Electron Microscopy (TEM) Analysis

**Figure 5:**
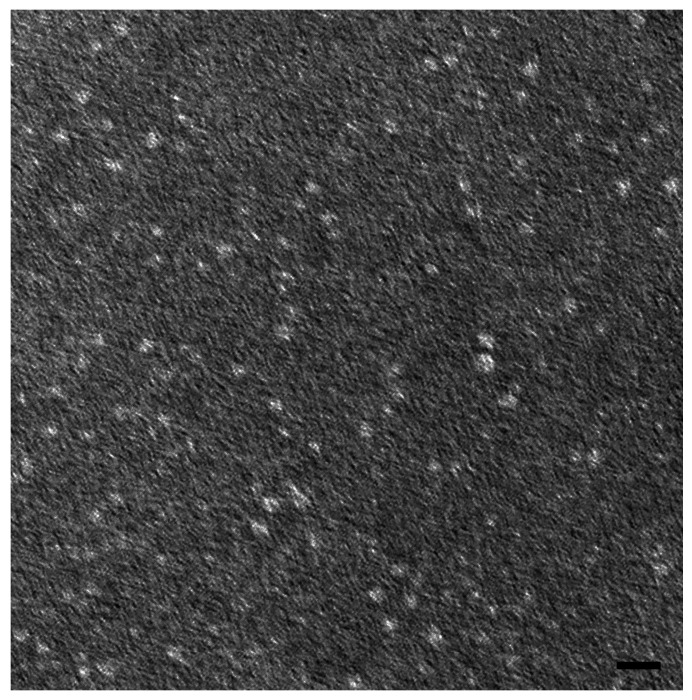
Microscopic observation of TPGS30ELP15 under TEM; scale bar 40 nm.

TEM investigation showed that the nanomicelles were spherical in shape and dispersed uniformly into the aqueous medium (Figure 5). This analysis confirmed that the binary mixture of surfactants produced a nanomicellar structure, and the particle sizes visualized by TEM were very similar and in agreement with the size obtained by DLS.

### 2.3. Physico-Chemical Stability of TPGS30ELP15 Formulation

Subsequent studies were performed to monitor the stability of curcumin when encapsulated in a nanostructure. Nanostructured systems are extensively documented in the literature as effective strategies to enhance the solubilization of lipophilic active ingredients in hydrophilic environment, while simultaneously protecting them from degradation phenomena. It is well known that curcumin undergoes structural alterations and degradation when exposed to both artificial and natural light, whether in the solid state or in solution [46,47].

**Figure 6:**
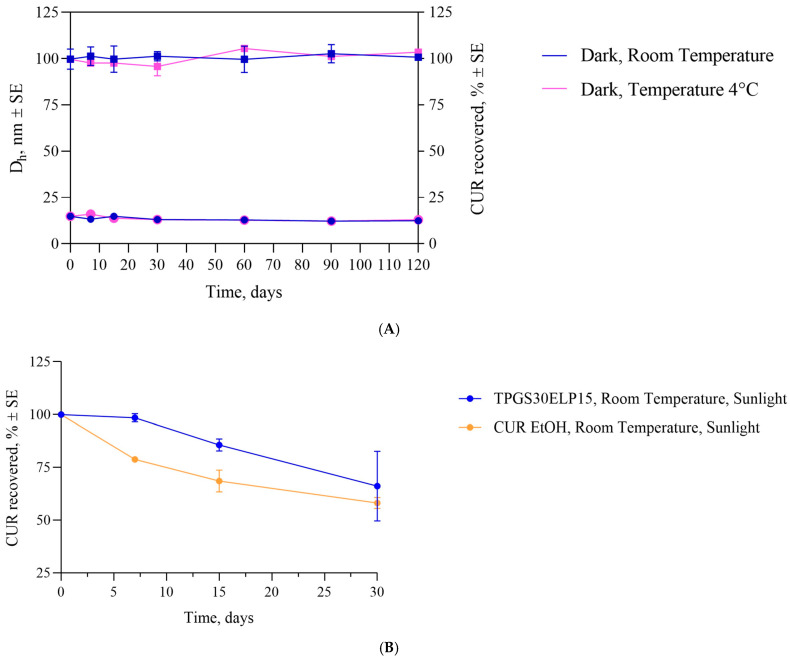
Time-dependent changes in size (•, D_h_) and recovered CUR percentage (■, CUR recovered) for the TPGS30ELP15 formulation stored at 4 °C and room temperature, both in the dark (**A**) and under light exposure (**B**) (n = 3).

As shown in Figure 6A, the formulation under study (TPGS30ELP15) demonstrated stability throughout the entire monitored period (120 days) at the two tested temperatures (4 °C and room temperature) when protected from light. The size of the nanostructured system and the percentage of recovered CUR remained nearly unchanged for 120 days at both room temperature and 4 °C.

To evaluate whether the nanostructured formulation could protect curcumin from light-induced degradation, the change in curcumin concentration was monitored over a 30-day period while the TPGS30ELP15 formulation was stored at room temperature under light exposure. An ethanolic solution of CUR at the same concentration as the nanomicellar formulation (CUR EtOH) was used as reference. The nanostructured formulation maintained its integrity for 7 days (CUR%: 98.42 ± 1.92%) after which the curcumin content decreased to 85.59 ± 2.84 and 66.15 ± 6.49% at 15 and 30 days, respectively. Conversely, in the case of the ethanolic solution, the amount of curcumin decreased to 78.75 ± 1.13% after just 7 days, reaching 58.14 ± 2.64% after 30 days (Figure 6B). These findings suggest that the nanomicellar structure provides only short-term protection against light-induced degradation.

### 2.4. Cytotoxicity over Time of the Selected CUR Nanomicellar Formulation on A375 Cells

**Figure 7:**
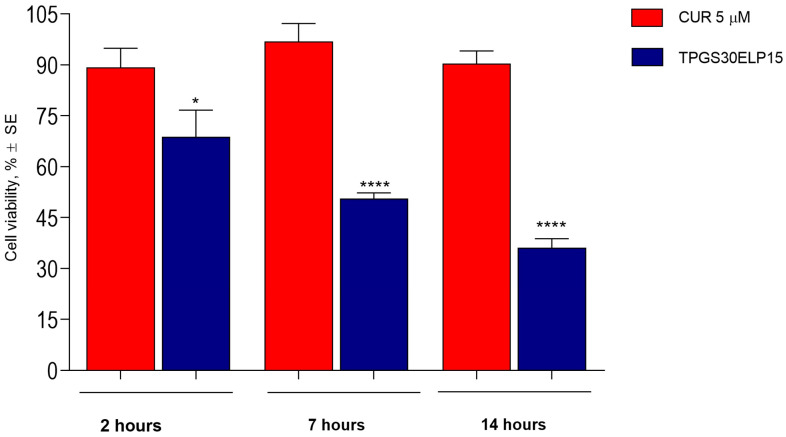
Time-dependent effect of the curcumin-loaded nanomicellar formulation, TPGS30ELP15, on A375 cells. Results are presented as means ± SEM from three independent replicates. * statistical significance *p* < 0.05; **** statistical significance *p* < 0.0001.

Time is a critical factor in drug action. The duration of target inhibition or the residence time of a drug molecule on its target can enhance its therapeutic effect. This study investigates the impact of exposure time (2, 7, and 14 h) on cytotoxicity and growth inhibition in A375 melanoma cells treated with CUR-loaded micelles. The results, illustrated in Figure 7, showed a notable time-dependent effect in inducing cell death: cell viability goes from 68.77 ± 7.88% after 2 h of contact to 50.56 ± 1.73% and 36.05 ± 2.28% after 7 and 14 h, respectively, with statistically significant differences compared to the reference (CUR 5 µM). CUR, a key component of turmeric, is known to significantly inhibit proliferation by arresting the cell cycle and by inducing apoptosis of human A375 melanoma cells in a dose- and time-dependent manner [48]. Liao and collaborators [48] demonstrated this effect by applying very high doses of CUR (2080 µM). Additionally, Zang et al. [49] calculated an IC50 value of 10 mM after 48 h of exposure from a dose–effect curve. In our study, we selected a low concentration of CUR to further highlight the ability of the selected formulation to better interact with cellular structures, thereby enhancing CUR’s efficacy. The data obtained support this approach.

### 2.5. Long-Term Toxicity Evaluation of CUR Nanomicellar Formulations on A375 and Balb/3T3 Clone A31 Cells

**Figure 8:**
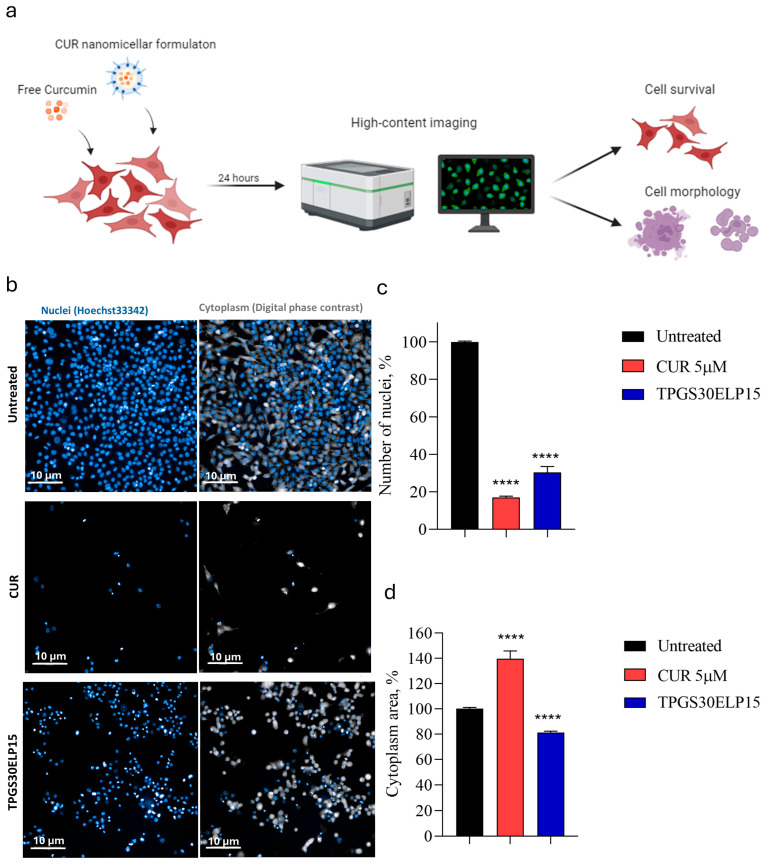
CUR and TPGS30ELP15 induce cell death by different pathways. (**a**) Illustration of the experimental workflow. (**b**) Representative images of A375 cells treated or not with CUR or TPG30ELP15 and analyzed 24 h after. Nuclei were stained with Hoechst 33,342 (Blue) and cytoplasm was reconstructed using Digital Phase Contrast (Gray). Number of nuclei (%) (**c**) and cytoplasmatic area (**d**) were measured by high-content confocal microscopy assays and expressed compared to the untreated counterparts. Data are expressed as mean ± SEM and were analysed using one-way ANOVA (n = 3; **** *p* < 0.0001).

Our study aimed at evaluating the long-term toxicity and the cell death induced by the nanomicellar formulation loaded with curcumin (TPGS30ELP15) in A375 cells. Cell viability was assessed by counting the nuclei and measuring the cytoplasmic area of A375 cells treated or not with either free curcumin (CUR) or TPGS30ELP15 at a concentration of 5 μM for 24 h (Figure 8a,b). The survival rate decreased by 85% and 70% in A375 cells treated with CUR and TPGS30ELP15, respectively (Figure 8c).

We also observed a 30% increase in the cytoplasmic area of cells treated with free CUR, indicative of cytoplasmic swelling and necrosis [50]. In contrast, treatment with TPGS30ELP15 resulted in a 20% decrease in the cytoplasmic area, suggesting apoptosis induction (Figure 8d).

**Figure 9:**
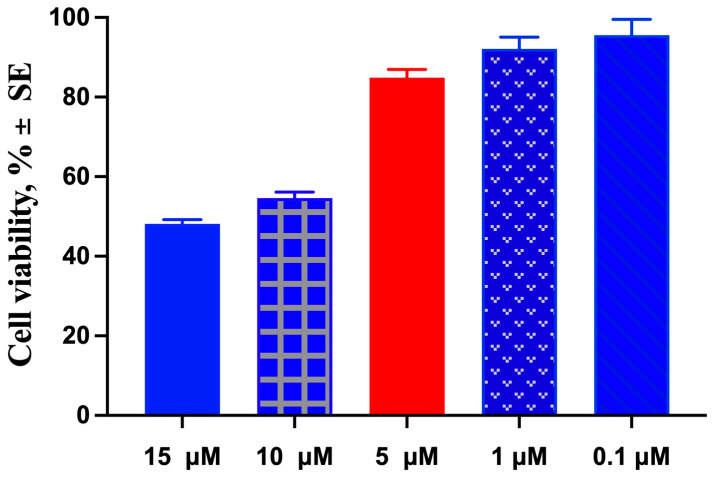
Percentage cell viability of Balb/3T3 clone A31 cells after treatments with TPGS30ELP15 at CUR concentration ranging from 0.1 to 15 μg/mL for 24 h.

Moreover, the cytotoxicity of TPGS30ELP15 was evaluated on Balb/3T3 clone A31 cells treated with the formulation for 24 h with CUR concentrations in the range 0.1–15 μM to demonstrate that the formulation under study is not toxic to healthy cells. The results are shown in the Figure 9, and it can be observed that after 24 h of treatment, the percentage of live cells was always above 50%. In particular, the concentration of CUR (5 μM) used for melanoma treatment with TPGS30ELP15 maintained a cell viability of 84.9± 2.08%, thus demonstrating that the formulation is not toxic to healthy fibroblasts (EC50 calculated from sigmoidal curve: 37.64 μM).

Taken together, these results underscore the significant impact of both free curcumin and TPGS30ELP15 in reducing melanoma cell viability. While free curcumin demonstrates a stronger cytotoxic effect (85% reduction) compared to TPGS30ELP15 (70%), it predominantly induces necrotic cell death, whereas TPGS30ELP15 promotes apoptosis. This distinction is reflected in the morphological changes observed post-treatment: CUR-treated cells exhibit cytoplasmic swelling, indicative of necrosis, while TPGS30ELP15-treated cells show a reduction in cellular volume, a hallmark of apoptosis.

A 30% increase in cytoplasmic area following CUR treatment suggests that necrosis is the primary mode of cell death. Necrosis, often resulting from acute damage, leads to the release of intracellular components into the extracellular space, potentially inciting inflammation and potentially harming surrounding tissues. While free CUR effectively decreases tumor cell viability, its tendency to provoke inflammatory responses could limit its clinical applicability despite its potent anti-cancer effects.

Conversely, TPGS30ELP15-treated cells exhibited a 20% decrease in the cytoplasmic area, indicative of apoptotic cell death. Apoptosis is a highly regulated process characterized by cellular shrinkage, chromatin condensation, and formation of apoptotic bodies, which are efficiently phagocytosed by neighboring cells without eliciting an inflammatory response [51]. This mechanism offers a therapeutic advantage as it minimizes collateral damage to healthy tissues and prevents inflammation. The ability of TPGS30ELP15 to induce apoptosis rather than necrosis suggests a superior safety profile, making it a promising candidate for further development as an anti-cancer therapy.

Curcumin’s broad spectrum of molecular targets, including thioredoxin reductase, cyclooxygenase-2 (COX-2), protein kinase C, 5-lipoxygenase (5-LOX), and tubulin, enables it to interfere with multiple cellular pathways involved in tumor growth and survival. Curcumin’s ability to activate apoptotic pathways and inhibit proliferative signaling is well-documented [52]. However, delivering curcumin via TPGS30ELP15 micelles appears to modulate these effects in a more selective manner, favoring apoptosis over necrosis.

TPGS, known for its selective cytotoxicity against tumor cells, enhances the therapeutic efficacy of curcumin by inducing apoptosis through multiple mechanisms. Acting as a reactive oxygen species (ROS) inducer, TPGS disrupts mitochondrial respiratory complex II, leading to DNA damage and oxidative stress. Additionally, TPGS downregulates anti-apoptotic proteins such as Survivin and Bcl-2 through the phosphorylation of protein kinase B (PKB or AKT), facilitating the activation of caspase-dependent programmed cell death pathways [32]. These mechanisms highlight the potential of TPGS30ELP15 as a highly effective delivery system that not only enhances curcumin’s bioavailability but also strengthens its pro-apoptotic effects, thereby improving its overall anti-cancer efficacy.

Our findings highlight the therapeutic potential of the curcumin-loaded nanomicellar formulation TPGS30ELP15 for melanoma treatment. Although free curcumin effectively reduces cell viability, its tendency to induce necrosis—and consequently trigger inflammatory responses—represents a significant drawback. In contrast, TPGS30ELP15 exhibits a more favorable therapeutic profile by predominantly inducing apoptosis, thereby reducing inflammation and minimizing damage to surrounding tissues.

## 3. Materials

The following materials were used: Curcumin (CUR, molecular weight 368.38 g/mol, Sigma-Aldrich, St. Louis, MO, USA), pharmaceutical grade d-α-Tocopherol Polyethylene Glycol Succinate (Vitamin E-TPGS, TPGS 1000, molecular weight 1513 g/mol, PMC ISOCHEM, Vert-Le-Petit, France, TPGS), Polyoxyl-35-castor oil (Kolliphor ELP, molecular weight 2500 g/mol, ELP, BASF, Ludwigshafen, Germany); WST-1 cell proliferation reagent (Roche Diagnostics GmbH, Mannheim, Germany); cell-permeant dye Hoechst 33,342 (Sigma-Aldrich, St. Louis, MO, USA). All other chemicals and solvents were of analytical grade. Ultrapure water was prepared using Milli-Q^®^plus apparatus (Millipore, Milan, Italy).

## 4. Methods

### 4.1. Design of Experiment (DoE) Study for the Selection of a Curcumin-Loaded Nanomicellar Formulation Based on a Binary Mixture of Surfactants

A suitable DoE study was settled to evaluate the impact of two chosen surfactants, TPGS and ELP, either individually or in combination, on two dependent variables: amount of solubilized curcumin (CUR) and cell viability on A375 melanoma cell lines. A full-factorial design with 3 × 3 quadrature points for the two independent variables was applied. This methodology enabled the identification of optimal conditions for the desired responses and provided deeper insights into the underlying mechanisms influencing the system. A first-order polynomial response surface was computed using MATLAB (version R2024b, MatWorks^®^, Natick, MA, USA) for the selected output quantities as a function of the two independent variables: X1 = [TPGS, mM] and X2 = [ELP, mM].

Models of varying complexity were tested to determine the best fit for the data:

- Quadratic Model: Z = β0 + β1TPGS + β2ELP + β3TPGSELP + β4TPGS^2^ + β5ELP^2^
- Linear Interaction Model: Z = β0 + β1TPGS + β2ELP + β3TPGSELP
- Simplified Linear Model: Z = β0 + β1TPGS + β2ELP

The models were evaluated using R-squared (R^2^) and adjusted R-squared (adj R^2^) values to assess the goodness of fit. Analysis of variance (ANOVA) was conducted to determine the significance of each model, using F-statistics and *p*-values. Response surface analysis was employed to visualize the effects of TPGS and ELP on the response variables. The response surfaces were generated using the fitted models selected to provide a graphical representation of the interactions between the surfactants and their impact on CUR solubilization and cell viability.

#### 4.1.1. Preparation of CUR-Loaded-Micelles

CUR micelles were prepared by melting two non-ionic surfactants (TPGS and ELP), either individually or in combination with different molar ratios, at 50 °C, for 1 h. Subsequently, an excess of CUR (5.45 mM when using a single surfactant and 8.14 mM for the mixture of the two) was added, and the mixture was stirred until a homogeneous blend was obtained. After water addition, stirring continued for 12 h and filtering through 0.20 µm pore size filters (0.20 μm RC Syringe filter, Phenomenex, Torrance, CA, USA) the unloaded drug, aggregates and other foreign particulates were removed and clear nanomicellar dispersions were formed. The composition of the nanomicellar dispersion is summarized in Table 3.

#### 4.1.2. Quantitative Analysis of Curcumin

The quantitative determination of solubilized curcumin in nanomicellar systems was carried out by HPLC analysis. The apparatus consisted of a Shimadzu LC-20AD system with a UV-VIS SPD-10A detector, a SIL10AD VP automatic injector (20 μL) and a C-R4A integration system (Shimadzu Italia s.r.l., Milan, Italy). The injection valve was a Rheodyne with a capacity of 20 µL, the column was a Luna-C_8_ (250 × 4.6 mm, Phenomenex, Torrance, CA, USA) with a particle size of 10 µm. The column was thermostated at 30 °C with a HPLC column temperature controller (ThermasphereTM, Phenomenex, Torrance, CA, USA). The mobile phase, running at an isocratic flow rate of 1.0 mL/min, consisted of acetonitrile:acetic acid 4% in a ratio of 55:45. Detection as performed at a wavelength of 424 nm, and the retention time under these conditions was 5 min. All solvents used were HPLC grade.

The CUR amount in the samples was determined by comparison with an external standard curve, which was linear within the studied concentration range of 0.0526–1.052 µg/mL (R^2^ = 0.9986). This standard curve was obtained by adding increasing amounts of the product to ethanol.

Nanomicellar formulations were filtered (0.20 µm pore size), diluted with ethanol, sonicated for 3 min, and centrifugated at 13,000 rpm for 7 min. The entrapment efficiency was calculated according to the following Equation (7):

(7) $$Entrapment\;efficiency\;\%=\frac{\;mass\;of\;CUR\;in\;nanomicelles}{mass\;of\;feed\;CUR\;in\;the\;mixture}\times 100$$

#### 4.1.3. Dynamic Light Scattering Analysis

The average hydrodynamic diameter (D_h_) and polydispersity index (PDI) of the formulations was determined by Dynamic Light Scattering (DLS) technique using Zetasizer Nano ZS (Malvern). Just before the DLS measurements, each sample was appropriately diluted with ultrapure water freshly filtered through 0.20 µm pore size filters to ensure that the concentration of micelles fell within the measurement intensity range of 5 × 10^4^ to 1 × 10^6^ counts per second. D_h_ and PI were measured at 25 °C with three runs for each sample, using an angle of 173° and a run time of 200 s.

#### 4.1.4. TEM Analysis

The morphological features (size and shape) of the nanomicelles in gelled mixtures were observed by TEM (JEOL 100 SX, model AMT XR80B, JEOL, Tokyo, Japan) equipped with a CCD camera. The samples were prepared using the following procedure: first, 10 μL of each sample was placed onto a copper grid with a formvar/carbon (200 mesh) membrane and left for 5 min, and after removing excess solution with filter papers, the copper grid was gently washed with drops of deionized water. Subsequently, the copper grid was negatively stained with 20 μL of uranyl acetate solution (2%, *w*/*v*) for 1 min at room temperature. The excess solution was removed with filter papers, and the sample was left to dry by air for 15 min. Then, it was examined under TEM, operating at an acceleration voltage of 100 kV.

#### 4.1.5. Cell Viability Assay

A375 and Balb/3T3 clone A31 cells were purchased from line American Type Culture Collection (Manassas, VA 20110, USA). All cells were cultured at 37 °C in 5% CO_2_ in Dulbecco-Modified Eagle’s Medium (DMEM). In case of A375 cells, the medium was supplemented with 10% fetal bovine serum (FBS), 1% sodium-pyruvate and 1% L-glutamine. For Balb/3T3 clone A31 cells the medium was supplemented with 10% fetal calf serum, 2% L-glutamine, 1% antibiotics and 0.1% antimycotic.

Cells were seeded in 96-CellCarrier Ultra plates (PerkinElmer, Hamburg, Germany) at a concentration of 1 × 10^5^ cells/mL and treated upon reaching about 90% confluence (24 h).

For the DoE analysis, A375 cells were treated with curcumin-loaded formulations for two hours. For the selected TPGS30ELP15 formulation, cell viability was assessed 2, 7 and 14 h post-treatment.

The formulations were diluted in culture medium to achieve a final CUR concentration of 5 µM. A 5 µM CUR solution was prepared by appropriately diluting a stock solution in DMSO with culture medium and used as a reference.

For non-neoplastic cells, Balb/3T3 clone A31 cells were treated for 24 h with varying concentrations of TPGS30ELP15 formulation diluted in growth medium to obtain CUR concentrations ranging from 0.1 to 15 μM.

Cells viability was assessed using the WST-1 assay [29].

### 4.2. Characterization of the Selected CUR Nanomicellar Formulation (TPGS30ELP15)

Size, amount of drug encapsulated, and EE% of the selected formulation, TPGS30ELP15, were determined using the methods above described.

To evaluate the interaction of CUR with the surfactants and assess its encapsulation inside the lipophilic core of micelles, ATR-FTIR spectra of curcumin powder (CUR), empty and CUR-loaded micelles after freeze-drying (Empty TPGS30ELP15-F and TPGS30ELP15-F, respectively) were recorded with an IR Cary 660 FTIR spectrometer (Agilent Technologies Santa Clara, CA, USA) using a macro-ATR accessory with a Zn/Se crystal. The empty micelles were prepared following the same method reported in the paragraph 3.1.1 without the addition of the drug. The spectra were measured in a range from 4000 to 500 cm^−1^, with 64 scans both for background and samples.

Thermal analysis of CUR, empty TPGS30ELP15-F, and TPGS30ELP15-F was performed using differential scanning calorimetry (DSC) with a DSC 6 calorimeter (PerkinElmer, Milan, Italy). Samples (1.5–2.0 mg) were sealed in flat-bottomed aluminum pans and heated at a constant rate of 5 °C/min under a nitrogen purge at 20 mL/min. The temperature range was set from 5 °C to 250 °C. Thermal profiles were recorded using Pyris Instrument Managing Software (Version 3.8, PerkinElmer, Milan, Italy) and analyzed with OriginPro^®^ software (Version 2018, OriginLab Corporation, Northampton, MA, USA)

NMR spectra of the TPGS30ELP15 formulation and the single components (CUR, TPGS and ELP) were measured on a JEOL JNM-ECZ500R spectrometer operating at 500 MHz for ^1^H (JEOL Italia S.p.A., Milan, Italy). All NMR spectra were referenced through the solvent lock (^2^H) signal according to IUPAC recommended method and the manufacturer’s protocols. Solvent suppression sequence employed was WATERGATE with perfect echo. All deuterated solvents used were purchased from Deutero (Deuteto GmbH, Kastellaun, Germany). CUR samples were freshly prepared and immediately analyzed.

The freeze-drying process took place under the following operating conditions: *freezing phase*—pressure 400 torr; temperature—38 °C; rate 0.6 °C/h with an additional freeze time of 120 min; *primary drying*—pressure 100 torr; temperature—38 to 0 °C; rate 2.1 °C/h; *secondary drying*: pressure 50 torr; temperature 0 to 25 °C; rate 5.0 °C/h followed by an additional drying step at 27 °C for 60 min.

Furthermore, the stability of TPGS30ELP15 formulation was monitored over time (7, 15, 30, 45, 60, 90, 120, 150, 180 days) at 4 °C and at room temperature in the dark. The amount of CUR remained in the formulation was quantified by HPLC while the size of micelles was determined by DLS analysis.

Moreover, the nanomicellar formulation was kept for 30 days in the natural light at room temperature. An ethanolic solution of CUR at the same concentration (reference) was subjected to the same conditions.

### 4.3. High-Content Imaging Analysis on A375 Cells

A375 cells were treated with TPGS30ELP15 nanomicellar formulations loaded with curcumin (CUR) at a concentration of 5 μM for 24 h. Then, cells were stained with the cell-permeant dye Hoechst 33,342 nucleic acid stain at concentration of 50 µg/mL in cell medium as previously described [53].

Images were acquired using Operetta CLS high-content imaging device (PerkinElmer, Hamburg, Germany) and analyzed with Harmony 4.6 software (PerkinElmer Hamburg, Germany). Imaging was performed at 20× magnification, capturing ~57 fields per sample in at least three technical replicates. Nanomicellar formulation toxicity assay was performed with the following building blocks: find nuclei > find cytoplasm > calculate morphology properties (area), by counting the number of nuclei 24 h post-treatment, as previously described [54].

### 4.4. Statistical Analysis

All data are expressed as mean ± standard error. Statistical differences were evaluated for two groups applying Student’s two-tailed unpaired *t*-test (GraphPad Prism Software, version 10, San Diego, CA, USA).

Statistical analyses for multiple groups were determined by one-way ANOVA test and Dunnett’s test for multiple comparisons. Statistical significance was defined as follows: * *p* < 0.05, ** *p* < 0.01, *** *p* < 0.001, **** *p* < 0.0001.

## 5. Conclusions

This study presents a novel nanostructured strategy for enhancing the therapeutic efficacy of curcumin in melanoma treatment through the development of autoassembling nanomicelles based on non-ionic surfactants (TPGS and ELP). Our findings demonstrate that TPGS30ELP15 formulation significantly improves curcumin’s solubility, stability, and anticancer activity, overcoming key limitations associated with its clinical application.

The optimized nanomicellar system enhances curcumin bioavailability while promoting preferential apoptosis over necrosis, thereby reducing the inflammatory responses typically associated with free curcumin treatment. This apoptotic mechanism provides a substantial therapeutic advantage, minimizing collateral damage to healthy tissues and improving safety in potential clinical applications.

Furthermore, the binary surfactant system employed in this study not only enables efficient curcumin encapsulation, but also exhibits intrinsic anticancer properties, reinforcing the overall efficacy of the formulation. The ability of TPGS to modulate oxidative stress and interfere with anti-apoptotic pathways further enhances the therapeutic potential of the nanomicelles.

From a translational perspective, our results highlight the potential of TPGS30ELP15 nanomicelles as a promising topical formulation for melanoma therapy. The formulation’s stability over time, coupled with its superior cytotoxic and pro-apoptotic effects on melanoma cells, supports its feasibility for further preclinical and clinical development. Future studies will focus on in vivo validation and optimization of drug delivery parameters to pave the way for safer and more effective melanoma treatments.

In conclusion, this work advances the field of nanotechnology-based drug delivery systems, providing a scalable and clinically relevant approach in enhancing the therapeutic efficacy of curcumin against melanoma.

## Figures and Tables

**Table 1:** Results of the characterization of the nanomicellar formulations used for design of experiment analysis: CUR solubilized, Dh, PDI, entrapment efficiency (EE), cell viability on A375 cell line. Mean ± SEM (n = 3).

Formulation	CUR Solubilized (mM)	Dh (nm)	PDI	EE (%)	Cell Viability (%)
TPGS20	2.85 ± 0.40	12.57 ± 0.92	0.441 ± 0.04	52.38 ± 7.43	72.02 ± 4.89
TPGS40	5.11 ± 0.31	12.67 ± 0.01	0.550 ± 0.04	93.85 ± 5.7	60.11 ± 7.04 **
ELP10	2.71 ± 0.68	13.79 ± 0.30	0.394 ± 0.01	49.72 ± 12.48	98.78 ±20.01
ELP20	3.34 ± 0.41	13.61 ± 0.21	0.611 ± 0.07	61.28 ± 7.52	98.43 ± 10.99
TPGS20ELP10	4.61 ± 0.65	12.76 ± 0.10	0.700 ± 0.04	56.70 ± 8.05	92.80 ± 6.84
TPGS20ELP20	6.17 ± 1.45	12.64 ± 0.09	0.507 ± 0.08	75.86 ± 17.87	89.07 ± 5.01
TPGS25ELP5	3.47 ± 0.10	11.47 ± 0.64	0.409 ± 0.03	42.69 ± 1.29	50.50 ± 4.85 ***
TPGS40ELP20	7.64 ± 1.72	12.66 ± 0.19	0.345 ± 0.01	93.86 ± 21.13	73.78 ± 4.05
TPGS40ELP10	5.57 ± 0.58	12.78 ± 0.29	0.470 ± 0.06	68.49 ± 7.87	71.08 ± 6.44

** Statistically significant difference (*p* < 0.01) vs. curcumin (CUR cell viability: 89.21 ± 5.64); *** Statistically significant difference (*p* < 0.001) vs. curcumin (CUR cell viability: 89.21 ± 5.64).

**Table 2:** Applied model characteristics and ANOVA analysis results for CUR solubilization and cell viability models.

Model Type	Test Number	Freedom Degrees	R^2^		Adj R^2^		F-Statistic		*p*-Value	
			CUR Solubilization	Cell Viability	CUR Solubilization	Cell Viability	CUR Solubilization	Cell Viability	CUR Solubilization	Cell Viability
Linear	9	3	0.935	0.625	0.913	0.5	42.9	5	0.00028	0.0527
Linear interaction	9	4	0.936	0.673	0.898	0.477	24.4	3.44	0.00206	0.109
Quadratic	9	6	0.941	0.700	0.843	0.200	9.62	1.4	0.0458	0.416

**Table 3:** Composition (mM) of the prepared nanomicellar dispersions.

Formulation	VitE-TPGS (mM)	ELP (mM)	Molar Ratio TPGS:ELP	Total Surfactant Content (mM)
TPGS20	20	0	-	20
TPGS40	40	0	-	40
ELP10	0	10	-	10
ELP20	0	20	-	20
TPGS20ELP10	20	10	2:1	30
TPGS20ELP20	20	20	1:1	40
TPGS25ELP5	25	5	5:1	30
TPGS40ELP20	40	20	2:1	60
TPGS40ELP10	40	10	4:1	50

## Data Availability

The original contributions presented in this study are included in the article/Supplementary Materials. Further inquiries can be directed to the corresponding author(s).

## Supplementary Materials

The following supporting information can be downloaded at: https://www.mdpi.com/article/10.3390/ph18030327/s1: Scheme S1. Keto-enol equilibrium of CUR (up). Expected main curcuminoids found in CUR (down); Figure S1. ^1^H NMR (500 MHz, 25 °C, DMSO-d_6_) spectrum of CUR (20 mM); Figure S2. ^1^H NMR (500 MHz, 25 °C, DMSO-d_6_) restricted spectral region of CUR (20 mM); Figure S3. ^1^H NMR (500 MHz, 25 °C, D_2_O) spectrum of ELP (10 mM); Figure S4. ^1^H NMR (500 MHz, 25 °C, D_2_O) spectrum of ELP (10 mM); Figure S5. ^1^H NMR WATERGATE (500 MHz, D_2_O, 25 °C) spectra of CUR, ELP, TPGS, and TPGS30ELP15; Figure S6. ^1^H NMR (500 MHz, D_2_O, 25 °C) spectra comparison of ELP without (1) and with WATERGATE water suppression (2); Figure S7. ^1^H NMR WATERGATE (500 MHz, D_2_O, 25 °C) spectrum of TPGS30ELP15.
