# Supplementary material for: Nanostructured Strategies for Melanoma Treatment—Part I: Design and Optimization of Curcumin-Loaded Micelles for Enhanced Anticancer Activity
Source: Pharmaceuticals (Basel). 2025 Feb 26;18(3):327. doi: 10.3390/ph18030327 (PMC11945392; doi:10.3390/ph18030327)
Supplement: Supplementary file 1 [file pharmaceuticals-18-00327-s001.zip › pharmaceuticals-3426866-supplementary.pdf]

Supporting Information

Nanostructured Strategies for Melanoma Treatment – Part I: Design and Optimization of Curcumin-Loaded Nanocarriers for Enhanced Anticancer Activity

Paganini V.[1,†], Cesari A.[2,†], Tampucci S.[1,3], Chetoni P.[1,3], Burgalassi S.[1,3], Lai M.[4], Sciandrone G[4]., Pizzimenti S.[1], , Bellina F.[2], Monti D[1,3].

[1]Department of Pharmacy, University of Pisa, Via Bonanno 33, Pisa (Italy)

[2]Department of Chemistry and Industrial Chemistry, University of Pisa, 56124 Pisa (Italy)

[3]Italian Inter-University Center for the Promotion of the 3Rs in Teaching and Research, University of Pisa, 56122 Pisa, Italy

[4]Retrovirus Center and Virology Section, Department of Translational Research and New Technologies in Medicine and Surgery, University of Pisa, 56100 Pisa (Italy)

[†] These authors contributed equally to this work

* Correspondence: silvia.tampucci@unipi.it

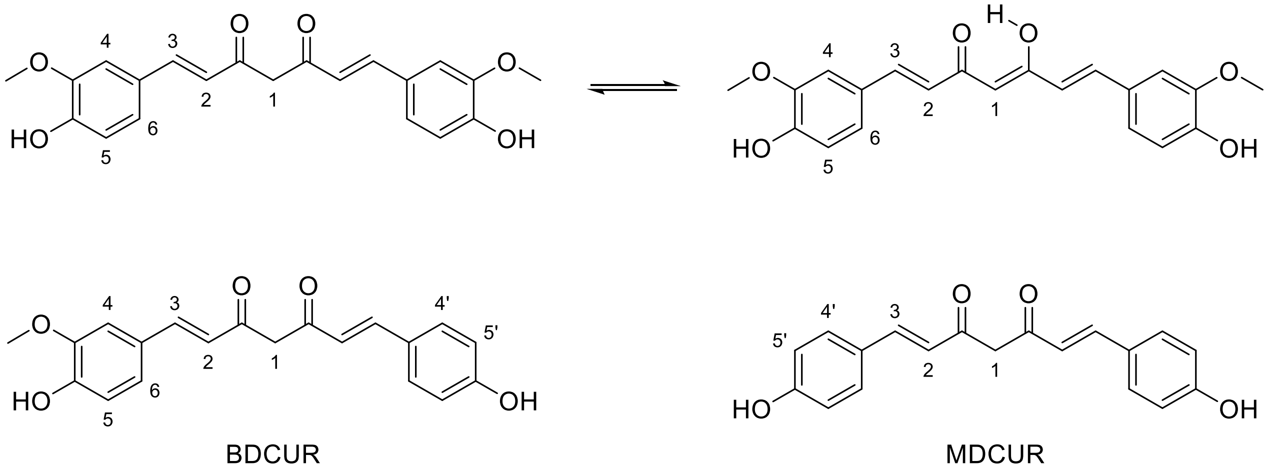

Scheme S1. Keto-enol equilibrium of CUR (up). Expected main curcuminoids found in CUR (down).

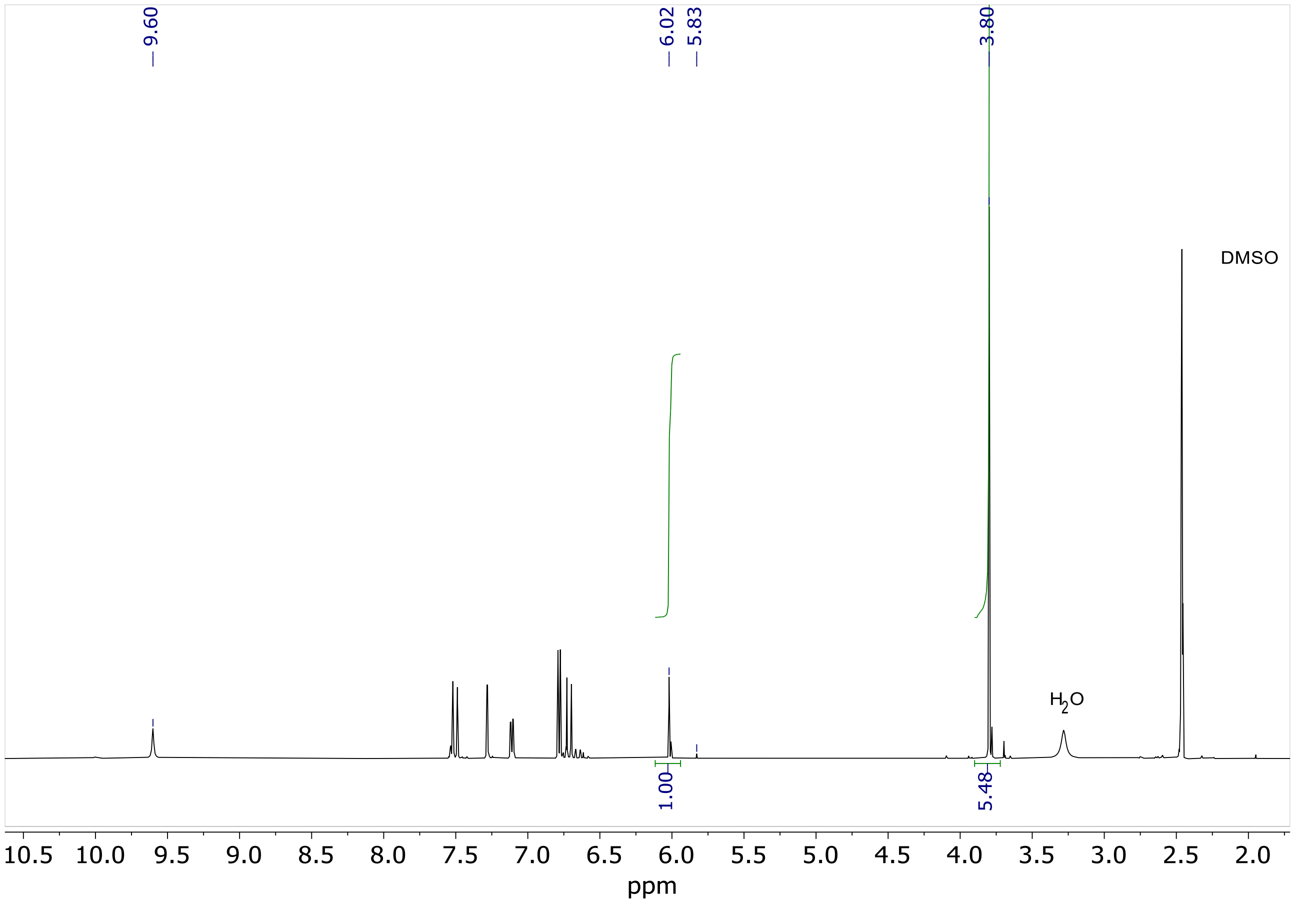

Figure S1. ^{1}H NMR (500 MHz, 25 °C, DMSO-d_6) spectrum of CUR (20 mM).

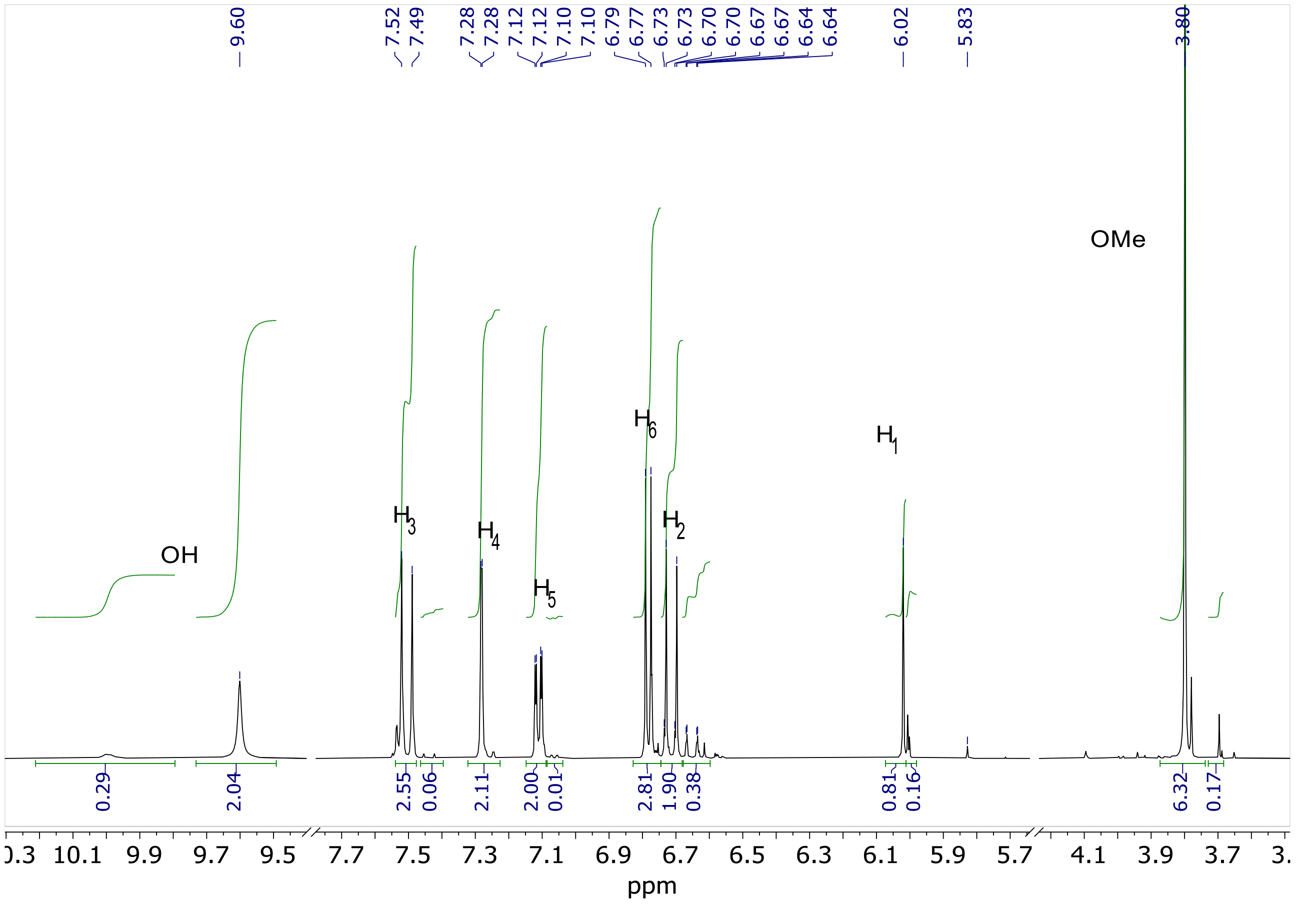

Figure S2. ^{1}H NMR (500 MHz, 25 °C, DMSO-d_6) restricted spectral region of CUR (20 mM).

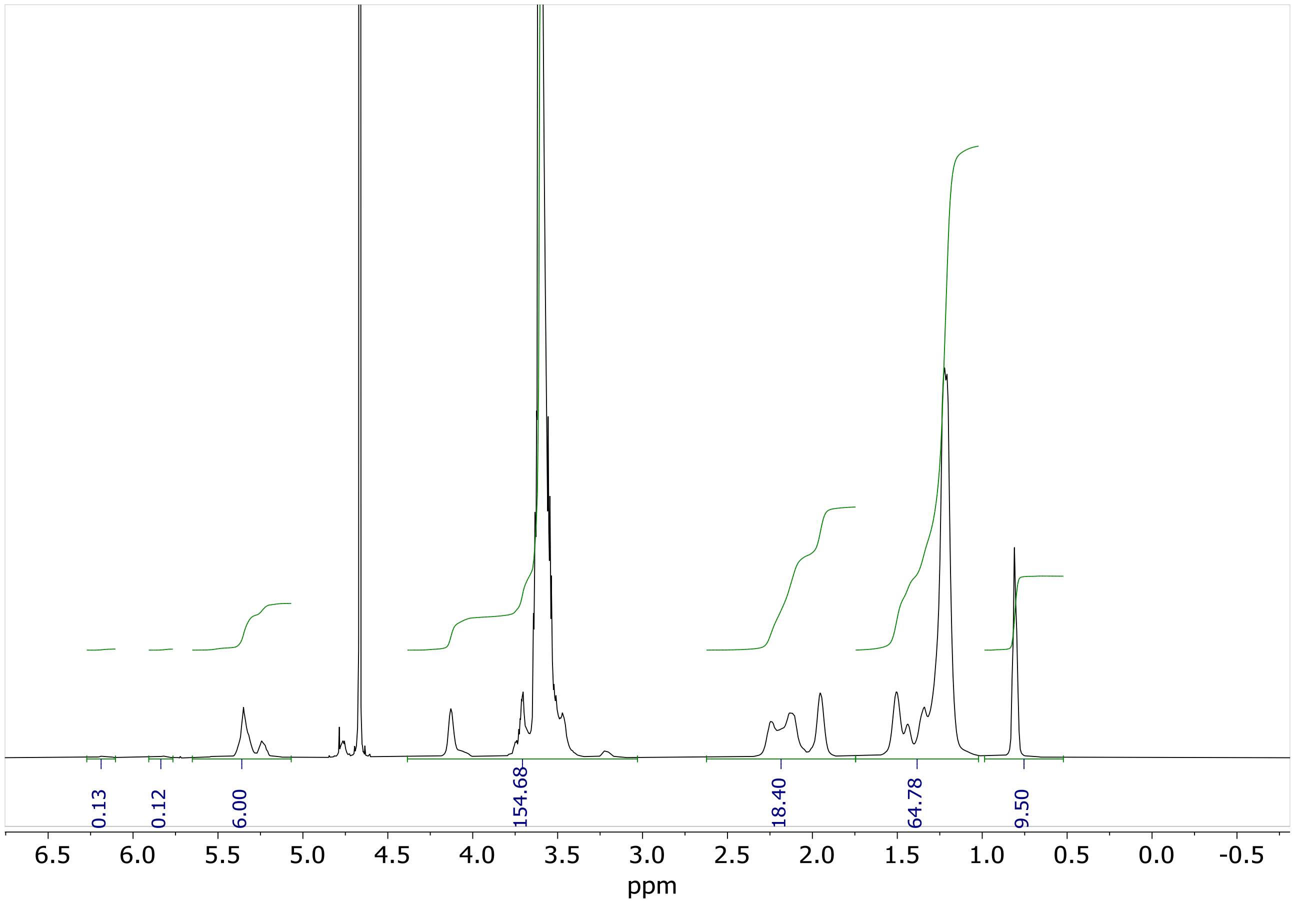

Figure S3. ^{1}H NMR (500 MHz, 25 °C, D_2O) spectrum of ELP (10 mM).

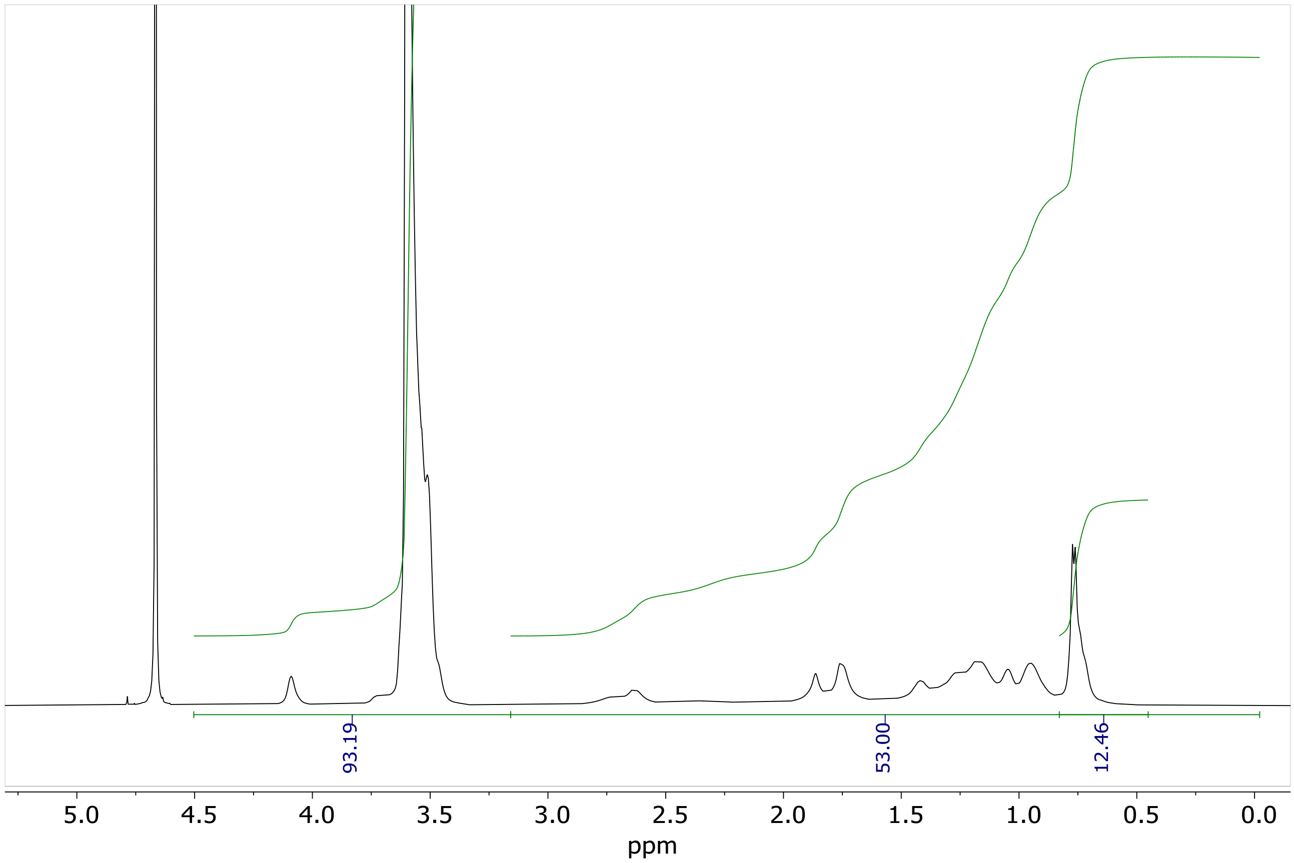

Figure S4. ^{1}H NMR (500 MHz, 25 °C, D_2O) spectrum of ELP (10 mM).

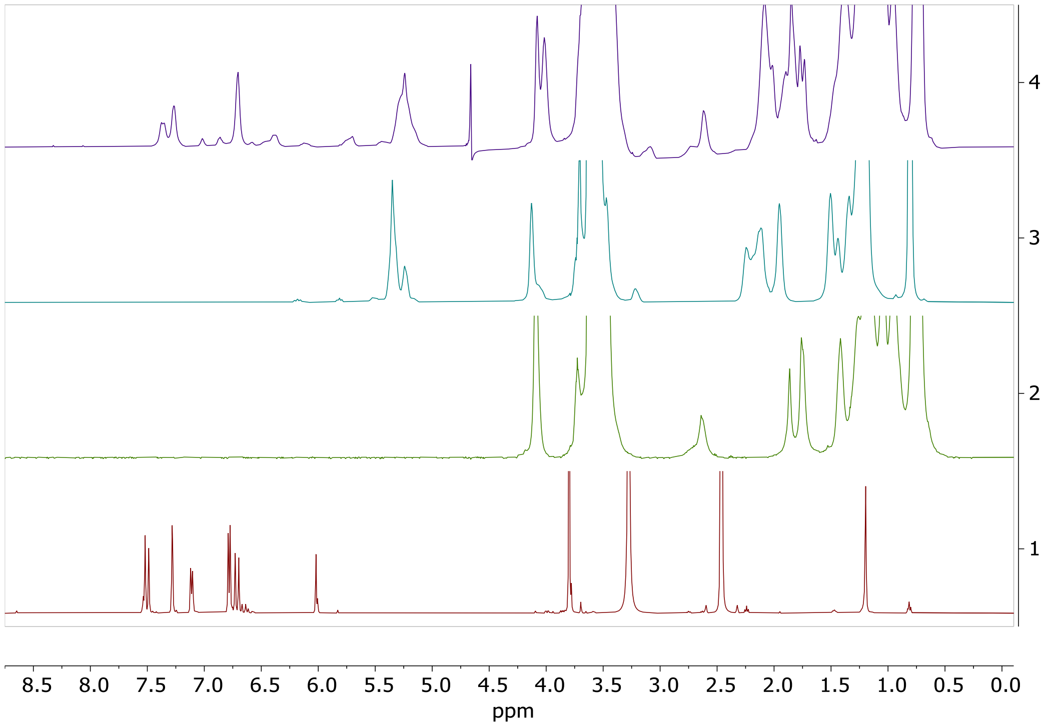

Figure S5. ^{1}H NMR WATERGATE (500 MHz, D_2O, 25 °C) spectra of CUR, ELP, TPGS, and TPGS30ELP15.

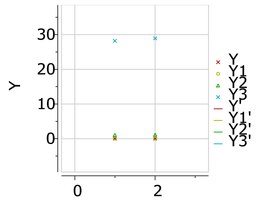

#	X(I)	Y(X)	Y1(X)	Y2(X)	Y3(X)
Model	ARR_DATA(I)	Integral(6.314, 5.992)	Integral(5.992, 5.689)	Integral(5.689, 5.086)	Integral(3.879, 3.274)
1	1.000	0.028	0.027	1.144	28.209
2	2.000	0.027	0.032	1.181	28.937

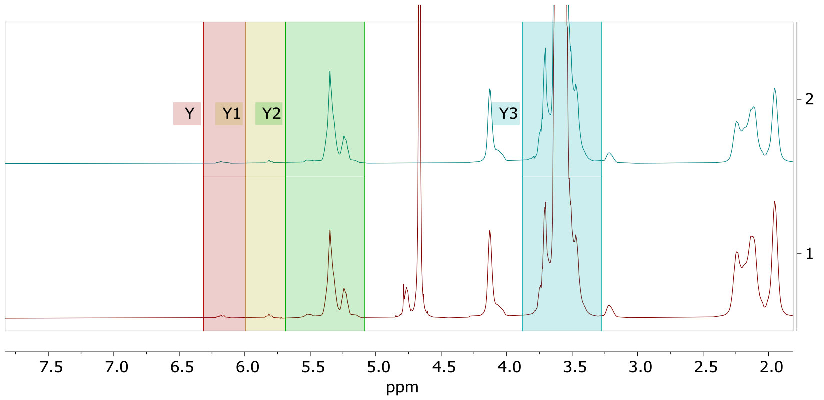

Figure S6. ^{1}H NMR (500 MHz, D_2O, 25 °C) spectra comparison of ELP without (1) and with WATERGATE water suppression (2).

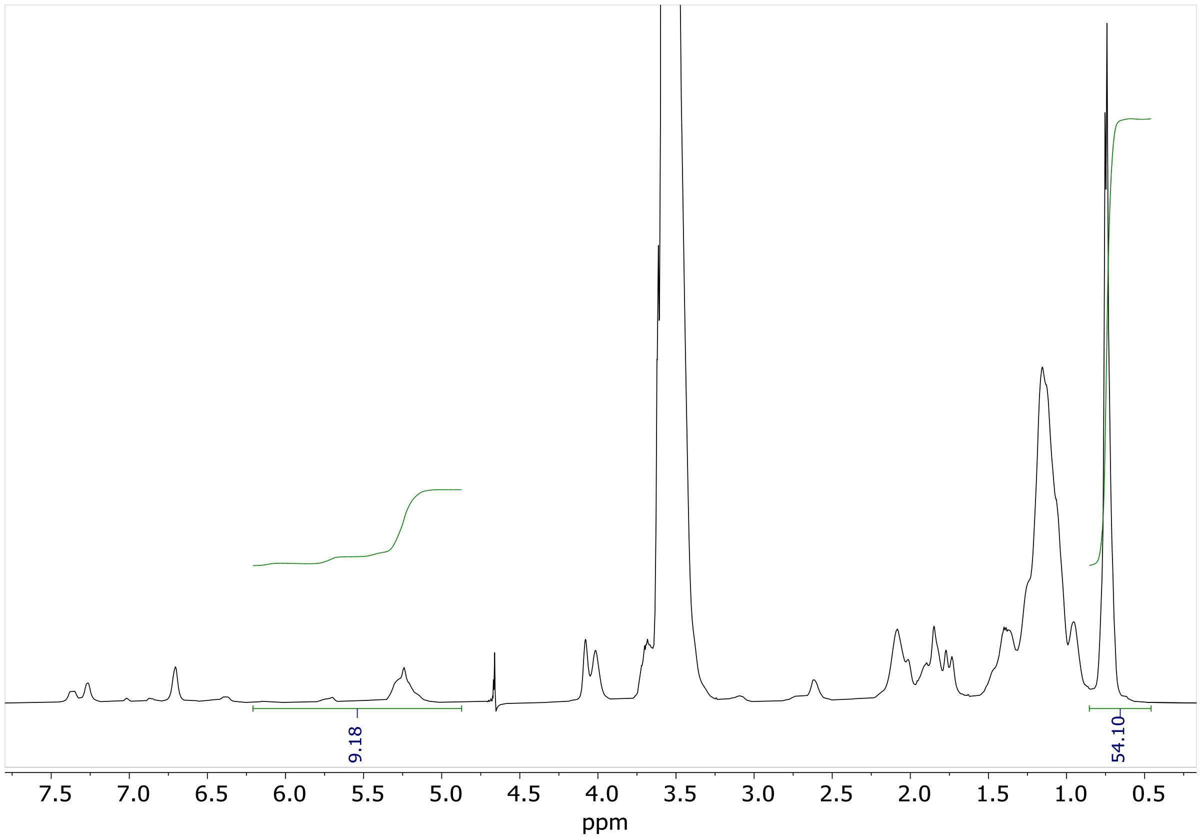

Figure S7. ^{1}H NMR WATERGATE (500 MHz, D_2O, 25 °C) spectrum of TPGS30ELP15.
